# Thrombin inhibitory activity of some polyphenolic compounds

**DOI:** 10.1007/s00044-013-0829-4

**Published:** 2013-10-16

**Authors:** M. Bijak, R. Ziewiecki, J. Saluk, M. Ponczek, I. Pawlaczyk, H. Krotkiewski, B. Wachowicz, P. Nowak

**Affiliations:** 1 Department of General Biochemistry, Faculty of Biology and Environmental Protection, University of Lodz, Pomorska 141/143, 90-236 Lodz, Poland; 2 Organic and Pharmaceutical Technology Group, Faculty of Chemistry, Wroclaw University of Technology, Wybrzeze Wyspianskiego 29, 50-370 Wroclaw, Poland; 3 Ludwik Hirszfeld Institute of Immunology and Experimental Therapy, Polish Academy of Sciences, Rudolfa Weigla 12, 53-114 Wroclaw, Poland

**Keywords:** Thrombin, Polyphenolic compounds, Flavonoids, Proteolytic activity, Inhibition

## Abstract

Thrombin, also known as an active plasma coagulation factor II, belongs to the family of serine proteases and plays a crucial role in blood coagulation process. The process of thrombin generation is the central event of the hemostatic process and regulates blood coagulant activity. For this reason, thrombin inhibition is key to successful novel antithrombotic pharmacotherapy. The aim of our present study was to examine the effects of the well-known polyphenolic compounds on the activity of thrombin, by characterization of its interaction with selected polyphenols using different biochemical methods and biosensor BIAcore analyses. Only six compounds, cyanidin, quercetin, silybin, cyanin, (+)-catechin and (−)-epicatechin, of all examined in this study polyphenols caused the inhibition of thrombin amidolytic activity. But only three of the six compounds (cyanidin, quercetin and silybin) changed thrombin proteolytic activity. BIAcore analyses demonstrated that cyanidin and quercetin caused a strong response in the interaction with immobilized thrombin, while cyanin and (−)-epicatechin induced a low response. Lineweaver–Burk curves show that used polyphenol aglycones act as competitive thrombin inhibitors. Our results suggest that polyphenolic compounds might be potential structural bases and source to find and project nature-based, safe, orally bioavailable direct thrombin inhibitors.

## Introduction

Serine proteases are a large group of enzymes that cleave peptide bonds in proteins. Mammalian genomes contain 2–4 % of genes which encode proteolytic enzymes (proteases) (Puente *et al*., 2005). Almost one-third of all proteases can be classified as serine proteases, named after the nucleophilic Ser residue at the active site (Hedstrom, 2002). In nature, the most abundant subfamily of serine proteases is chymotrypsin-like proteases (Rawlings *et al*., 2012). Occurring in all chymotrypsin-like serine proteases a conserved active center is located inside the molecule and contains amino acid residues of His 57, Asp 102 and Ser 195 (assuming chymotrypsin numbering), which are called the catalytic triad (Hedstrom, 2002).

Thrombin, also known as an active plasma coagulation factor II, belongs to the family of serine proteases and plays a crucial role in the blood coagulation process (Crawley *et al*., 2007). The process of thrombin generation is the central event of the hemostatic process, and regulates blood coagulant activity (Mann *et al*., 2006; McMichael, 2012). Thrombin is responsible for the second phase of blood coagulation process/cascade, where thrombin generated on TF-bearing cells activates blood platelets and also stimulates back other plasma coagulation factors (FXI, FVIII, FV) on the platelet’s surface (Hoffman and Monroe, 2007). Thrombin also converts the soluble fibrinogen into the insoluble fibrin clot (Wolberg, 2007) and stabilizes the clot by activation of transglutaminase factor XIII (Bijak *et al*., 2013a; Muszbek *et al*., 1999) and the thrombin activatable fibrinolysis inhibitor (TAFI) (Bajzar, 2000). The important role of thrombin in hemostasis and thrombosis processes is associated with cardiovascular diseases, which are almost half of the death causes in economically developed countries. The evidence for the increased production and in vivo action of thrombin is invariably found in the plasma of individuals at high risk for clinically significant venous or arterial thromboembolic disease (Brummel-Ziedins *et al*., 2005; Eichinger, 2008; Ofosu, 2006). The increased production and action of thrombin may even be stronger in persons with deep vein thrombosis and/or pulmonary embolism, acute coronary syndrome, myocardial infarction (Smid *et al*., 2011) or ischemic stroke (Faber *et al*., 2003). In view of the important role ascribed to thrombin in the establishment and progression of both venous and arterial thrombosis, thrombin inhibition is the key for novel, successful antithrombotic pharmacotherapy (Bijak and Bobrowski, 2010).

Researches carried out in the last years provide evidence that polyphenol compounds are able to inhibit the activity of many enzymes including serine proteases (Cuccioloni *et al*., 2009a).

In our in vitro previous studies, we have shown that polyphenol-rich extracts from black chokeberry and grape seeds have anticoagulant (Bijak *et al*., 2011) and antithrombin (Bijak *et al*., 2013b) properties.

The aim of our present study was to examine the effects of the well-known polyphenolic compounds on the activity of thrombin, the most important serine protease in plasma hemostasis, by characterization of its interaction with selected polyphenols using different biochemical methods and biosensor BIAcore analyses.

## Materials and methods

### Reagents

Thrombin from human plasma (T7009), bovine serum albumin (BSA), dimethyl sulfoxide (DMSO) and polyphenol compounds, such as 4-hydroxyphenylacetic acid gallic acid, ferulic acid, caffeic acid, chlorogenic acid, coumaric acid, resveratrol, cyanin, cyanidin, (+)-catechin, (−)-epicatechin, procyanidin B2, naringenin, naringin, hesperetin, hesperidin, quercetin, rutin, genistein and silybin, were obtained from Sigma-Aldrich Chemical Co. (St. Louis, MO, USA). Frozen human plasma obtained from whole blood collected into 0.32 % sodium citrate was purchased from the Regional Center for Transfusion Medicine in Lodz (Poland). Chromogenic substrate S-2238 was purchased from Chromogenix (Italy). Sensor chips CM5, amine coupling kit containing *N*-hydroxysuccinimide (NHS), 1-ethyl-3-(3-dimethylaminopropyl) carbodiimide hydrochloride (EDC) and ethanolamine hydrochloride were from BIAcore (Uppsala, Sweden). All other chemicals were of analytical grade or highest quality available products.

### Isolation of fibrinogen

Fibrinogen (fg) was isolated from citrated human plasma by the cold ethanol precipitation technique described by Doolittle et al. (1967). Its concentration was determined spectrophotometrically at 280 nm using an extinction coefficient (*A* = 1.55 OD for 1 mg/ml concentration of fibrinogen). Fibrinogen obtained by this method always contains a small amount of factor XIII (fibrin stabilizing factor).

### Blood platelet isolation

Blood samples in 0.32 % sodium citrate from healthy volunteers without cardiovascular disorders were collected, untreated with antiplatelet drugs. To obtain platelet-rich plasma (PRP), blood was immediately centrifuged (200×*g*, 10 min, RT). Platelets were isolated from PRP using BSA–Sepharose 2B gel filtration method according to Walkowiak et al. (2000).

The study was performed under the guidelines of the Helsinki Declaration for Human Research and approved by the Committee on the Ethics of Research in Human Experimentation at the University of Lodz (KBBN-UL/II/21/2011).

### Thrombin sample preparation

Human thrombin (initial concentration: 17.6 nM in 50 mM TBS, pH 7.4) was preincubated with polyphenolic compounds (4-hydroxyphenylacetic acid, gallic acid, ferulic acid, caffeic acid, chlorogenic acid, coumaric acid, resveratrol, cyanin, cyanidin, (+)-catechin, (−)-epicatechin, procyanidin B2, naringenin, naringin, hesperetin, hesperidin, quercetin, rutin, genistein and silybin) at the concentration range of 0.1–1,000 μM by 10 min at 37 °C. In these preparations, to nine volumes of thrombin one volume of polyphenolic compounds was added (final thrombin concentration was 15.8 nM). All tested compounds were dissolved in 50 % DMSO to the initial concentration of 10 mM; other solutions of compounds were also prepared in 50 % DMSO (prepared in 50 mM TBS, pH 7.4). The final concentration of DMSO in thrombin samples was 5 %. To prepare thrombin control samples, the same volume of solvent (50 % DMSO prepared in 50 mM TBS, pH 7.4) was added as in the case of the compound volume and warmed for 10 min to 37 °C.

### Determination of amidolytic activity of thrombin

The activity of human thrombin was determined by measuring the hydrolysis of chromogenic substrate D-Phe-Pip-Arg-pNA (Lottenberg *et al*., 1982; Sonder and Fenton, 1986). The absorbance measurements were performed at 415 nm using a 96-well microplate reader. To each reaction well, 40 μl of 3 mM chromogenic substrate was added. To initiate the chromogenic reaction, 280 μl of control thrombin (without tested compounds) or thrombin after preincubation with a polyphenolic compound to every reaction well in the same moment was added. The absorbance value was monitored every 12 s for 10 min. The maximal velocity of the reaction (*V*_max_, Δ*m* OD/min) for each absorbance curve was determined. IC_50_ value (parameter) for every polyphenolic compound from inhibition curves was estimated.

### The measurement of thrombin-induced fibrinogen polymerization

Polymerization of fibrin was monitored at 595 nm using a 96-well microtiter plate reader. To each reaction well of the microtiter plate, 100 μl of fibrinogen (3 mg/ml) in 50 mM TBS and 5 mM CaCl_2_, pH 7.4, were added. To initiate the polymerization reaction in all reaction wells, 200 μl of thrombin control mixture or thrombin solution preincubated with polyphenolic compounds (final concentration of thrombin—10.4 nM) was added. Thrombin-catalyzed fibrinogen polymerization was monitored every 12 s for 20 min at 37 °C. The maximal velocity of the polymerization process (*V*_max_, Δ*m* OD/min) for each absorbance curve was determined (Nowak *et al*., 2007).

### SDS-PAGE analysis

To 50 μl of fibrinogen solution (3 mg/ml in 50 mM TBS, 5 mM CaCl_2_), 100 μl of control thrombin or thrombin mixture preincubated with polyphenolic compounds (final concentration of thrombin—10.4 nM) was added. The reactions incubated at 37 °C were stopped after 5, 15 and 30 min by adding 150 μl of lysis buffer (0.125 M Tris/HCl, 4 % SDS, 8 M urea, 10 % β-mercaptoethanol, pH 6.8). Samples were subjected to SDS-PAGE (polyacrylamide concentration—7.5 %) using Mini-Protean Electrophoresis Cell (Bio-Rad, Hercules, CA). Proteins were stained with Coomassie Brilliant Blue R250 (CBB).

### The measurement of thrombin-induced platelet aggregation

The platelet aggregation was measured by turbidimetric method (Saluk-Juszczak *et al.*, 2007) using dual-channel Chrono-log optical aggregometer (Chronolog, USA). The platelet suspension isolated by BSA–Sepharose 2B gel filtration method was diluted by modified Tyrode’s buffer (127 mM NaCl, 2.7 mM KCl, 0.5 mM NaH_2_PO_4_, 12 mM NaHCO_3_, 5 mM HEPES, 5.6 mM glucose, pH 7.4) (Saluk-Juszczak *et al.*, 2008), to obtain the final platelet suspensions of 1.5 × 10^5^/μl. Platelet suspensions were pre-warmed at 37 °C with stirring. After 5 min the control thrombin solution or thrombin mixture preincubated with polyphenolic compounds (final concentration of thrombin—2.4 nM) was added, and aggregation of platelets was measured for 10 min. The aggregometer was calibrated every time (100 % aggregation) on Tyrode’s buffer with the appropriate concentration of each polyphenolic compound. The final concentration of DMSO in platelets samples were 0.77 %.

### Studies of thrombin interaction using a BIAcore system

The biosensor assays were performed using the BIAcore 1000 biosensor system. All biosensor analyses were performed with a phosphate-buffered saline (PBS), pH 7.4, as a running buffer. The polyphenolic compounds, as analytes, were diluted in PBS (final concentration of used polyphenolic compounds was 50, 100, 250, 500 and 1,000 μM).

The immobilization of thrombin on a biosensor carboxylmethyl dextran surface was performed according to the BIA applications Handbook (BIAcore, 1994). The process of protein immobilization was performed on a sensor chip CM5 surface by the positively charged functional groups of protein amino acids. The immobilization process consisted of four steps: preconcentration, activation, ligand immobilization and deactivation of the residual NHS esters. As a working buffer PBS with a constant flow rate of 5 μl/min was used. The temperature during the whole experiment was also constant and was set to 25.0 °C. The preconcentration step was started with preparation of different thrombin solutions by dissolving 5 μl thrombin solution (2.0 mg/ml deionized H_2_O) in 100 μl of different 50 mM acetic buffers (pH values: 4.0, 4.5, 5.0, 5.5 and 6.0, respectively). Each of these solutions (10 μl) was injected into an empty sensor chip channel. Acetate buffer which gave the highest detector response (50 mM, pH 6.0) was used for ligand immobilization The activation of carboxylated dextran surface was carried out with a mixture consisting of 25 μl of 0.1 M NHS and 150 μl of 0.2 M EDC, both dissolved in deionized H_2_O. 35 μl of the activation mixture was injected into an empty sensor channel at a flow rate of 5 μl/min. The amount of injected activation mixture was modified, to regulate the amount of immobilized ligand. To immobilize the ligand, thrombin was dissolved in deionized H_2_O to a final concentration of 2 mg/ml, and then 5 μl of this solution was added to 100 μl of acetic buffer chosen in the preconcentration stage of the experiment. 35 μl of mixture of thrombin in acetic buffer was injected immediately after activation of the sensor chip surface. To deactivate the rest of non-bonded carboxylmethyl dextran surface, 100 μl of 1 M ethanolamine hydrochloride solution, pH 8.5, and then 100 μl of 0.5 M NaCl solution were injected to the channel.

The conditions of the latter experiments were established by numerous pre-tests. The assessed parameters included: the buffer flow rate, the volume of analyte injection, the concentration of analytes, types and concentration of regenerators. Every 10 s before injection of each of the examined polyphenols, the detector baseline was measured. For each injection of analyte solution, the volume used was 100 μl. After injection of analyte was completed, the dissociation step occurred and the level of the interaction ligand–analyte was measured. During dissociation, the particles non-covalently bound to the ligand were washed out from the working channel. The solutions of 0.1 M NaOH and 0.1 M HCl were chosen for regeneration of the immobilized sensor channel, due to their good regeneration efficiencies and non-destructive influence on thrombin activity.

Shortly, the process of analysis in a channel with immobilized ligand and afterward regeneration of the channel, flow rate 10 μl/min, contained the following steps:

1. PBS injection, 900 s.
2. Polyphenol (analyte) injection, 600 s.
3. Dissociation (PBS injection), 200 s.
4. NaOH injection, 600 s.
5. PBS injection, 60 s.
6. HCl injection, 600 s.
7. PBS injection, 900 s.
8. Reading the detection level (resonance units, RU).

The output, a signal of BIAcore system, was presented in sensorgrams and measured in RU, where 1,000 RU is equal to 1 ng of an analyte mass bound per 1 mm^2^ (Fivash *et al*., 1998). Using BIAevaluation 3.1 software, the association rate (*k*_a_), the dissociation rate (*k*_d_) and the equilibrium constants (*K*_A_ and *K*_D_) were determined from sensorgrams for all used concentrations of analyte. To do these calculations, the formulas presented below were used:

the association rate

$$\frac{{{\text{d}}[AB]}}{{{\text{d}}t}} = k_{\text{a}} \cdot [A] \cdot [B];$$

the dissociation rate

$$- \frac{{{\text{d}}[AB]}}{{{\text{d}}t}} = k_{\text{d}} \cdot [AB];$$

the equilibrium constants:

$$K_{\text{A}} = \frac{[AB]}{[A] \cdot [B]} = \frac{{k_{\text{a}} }}{{k_{\text{d}} }}$$

$$K_{\text{D}} = \frac{[A] \cdot [B]}{[AB]} = \frac{{k_{\text{d}} }}{{k_{\text{a}} }}.$$

### Analysis of thrombin inhibition parameters

Thrombin was incubated with polyphenol compounds at IC_50_ concentration at 37 °C. After 10 min, 280 μl of thrombin control (without tested compounds) or thrombin preincubated with polyphenol compounds was added to reaction well containing, respectively, 40 μl of 1.5, 3, 4.5 and 6 mM chromogenic substrate (final concentrations of chromogenic substrate was 187.5, 375, 562.5 and 750 μM respectively). Absorbance was monitored every 12 s for 10 min in a 96-well microplate reader. The velocity of reaction was expressed as the increase in product (pNA) over time (∆ μmol/min) using a computer program Mikcroplate Manager^®^ 8 and the extinction coefficient of *p*-nitroaniline. (*ε* = 8,270/M/cm). Then, the Lineweaver–Burk (1934) curves for thrombin in the presence and in the absence of polyphenol compounds were plotted. The Lineweaver–Burk equation, which is a transformation of the Michaelis–Menten model, looks as follows:

$$\frac{1}{V} = \frac{{K_{\text{m}} }}{{V_{\hbox{max} } }} \cdot \frac{1}{[S]} + \frac{1}{{V_{\hbox{max} } }}$$

### Statistical analysis

The statistical analysis was performed using StatSoft Inc. “Statistica” v. 6.0. All the values in this study were expressed as mean ± SD. Results were analyzed under the account of normality with Shapiro–Wilk test and equality of variance with Levene test. The significance of differences between the values was analyzed depending on the Levene test by ANOVA followed by Tukey multiple comparisons test or Kruskal–Wallis test. A level *p* < 0.05 was accepted as statistically significant.

## Results

### Polyphenolic compounds effect on thrombin amidolytic activity

Only six compounds: cyanidin, quercetin, silybin, cyanin, (+)-catechin and (−)-epicatechin, of all examined polyphenols, caused the inhibition of thrombin amidolytic activity (Table 1). It was observed that these six compounds in a dose-dependent manner decreased the initial velocity of chromogenic substrate hydrolysis. The thrombin inhibition by the polyphenolic compound was expressed as IC_50_ value—the concentration of a polyphenol needed to inhibit 50 % of thrombin amidolytic activity. The strongest inhibitory effect was demonstrated by cyanidin and quercetin (IC_50_ for cyanidin at 0.25 μM and for quercetin 1.5 μM at 375 μM of substrate concentration). The six polyphenols manifesting inhibitory effect on thrombin amidolytic activity were selected for the next steps of the study.

**Table 1:** The effect of polyphenolic compounds on the amidolytic activity of human thrombin

Compound	IC_50_ (Μm)
Cyanidin	0.25
Quercetin	1.5
Silybin	25
Cyanin	75
(+)-Catechin	125
(−)-Epicatechin	150
4-Hydroxyphenylacetic acid	–
Gallic acid	–
Caffeic acid	–
Chlorogenic acid	–
Ferulic acid	–
Coumaric acid	–
Resveratrol	–
Naringenin	–
Naringin	–
Hesperetin	–
Hesperidin	–
Procyanidin B2	–
Genistein	–
Rutin	–

Thrombin was incubated with polyphenolic compounds (at the concentration range of 0.1–1,000 μM). The absorbance value was monitored for 10 min. IC_50_ (at 375 μM substrate concentration) was determined using inhibition curves. Mark “–” means no inhibitory effect on amidolytic activity of thrombin

### Polyphenolic compounds effect on thrombin proteolytic activity

**Fig. 1:**
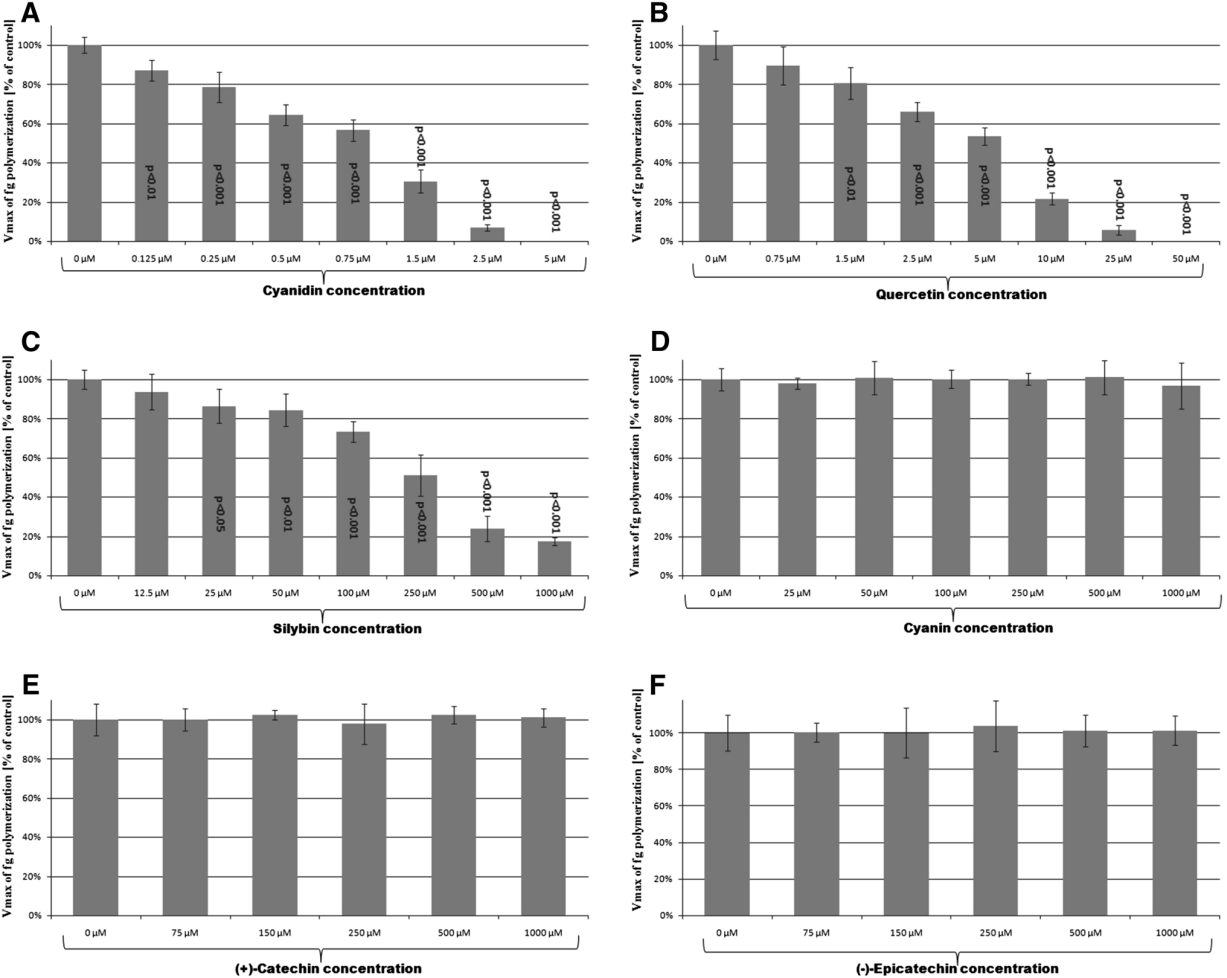
The effect of polyphenolic compounds [cyanidin, quercetin, silybin, cyanin, (+)-catechin and (−)-epicatechin] on the rate of thrombin-induced fibrinogen polymerization. Thrombin was preincubated with each if the polyphenolic compounds at the selected concentrations, at 37 °C for 10 min. Thrombin-catalyzed fibrinogen polymerization was monitored for 20 min, as the change of turbidity at 595 nm. The results are expressed as % of maximal velocity *V*_max_ of fg polymerization of the control samples (thrombin without tested polyphenols). Data represent mean ± SD of 12 independent experiments done in duplicates

**Fig. 2:**
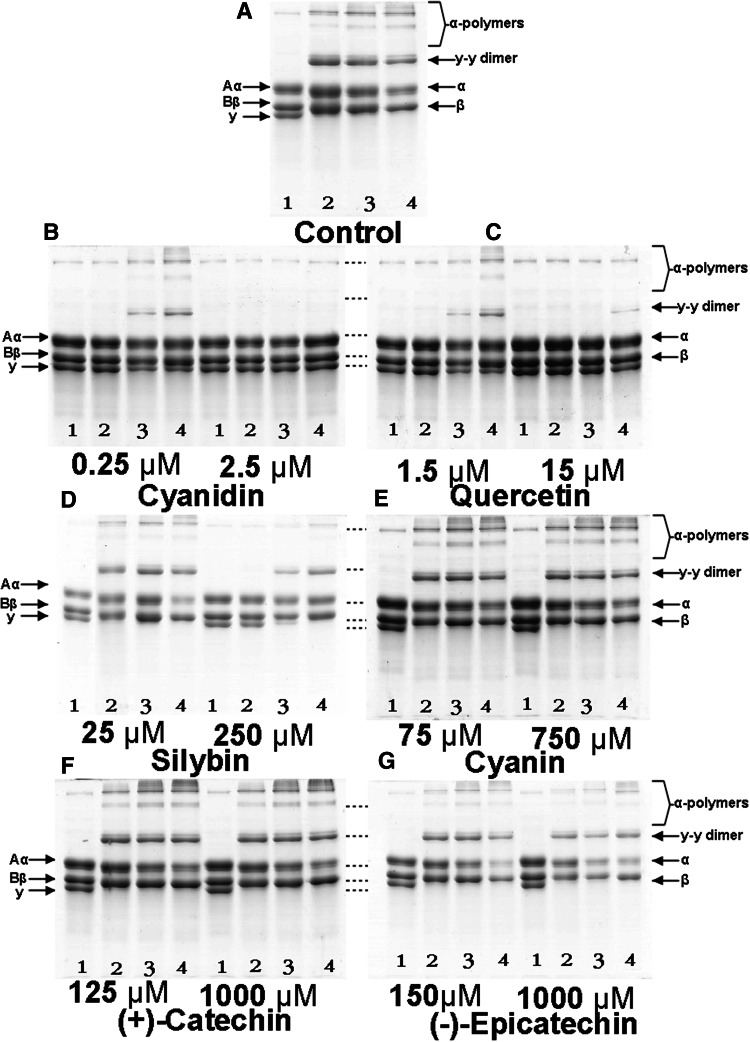
The effect of polyphenolic compounds [cyanidin, quercetin, silybin, cyanin, (+)-catechin and (−)-epicatechin] on thrombin-induced cross-linked fibrin formation, after treatment of fibrinogen (containing factor XIII). 100 μl of control thrombin or preincubated with polyphenols was mixed with 50 μl of fibrinogen (3 mg/ml), and, after the specified time, 150 μl of Laemmli sample buffer containing 8 M urea and 10 % β-mercaptoethanol was added to digest the mixture. Proteins were separated on 7.5 % SDS-PAGE gel and staining with Coomassie Blue R250. Positions of fibrinogen chains (Aα, Bβ and γ) and the cross-linked fibrin chains (α, β, γ–γ dimer and α-polymers) are indicated. **a** Control thrombin, **b** thrombin preincubated with cyanidin (0.25 and 2.5 μM), **c** thrombin preincubated with quercetin (1.5 and 15 μM), **d** thrombin preincubated with silybin (25 and 250 μM), **e** thrombin preincubated with cyanin (75 and 750 μM), **f** thrombin preincubated with (+)-catechin (125 and 1,000 μM), **g** thrombin preincubated with (−)-epicatechin (150 and 1,000 μM). Lane 1–4 reaction mixtures stopped after 0 s, 5, 15 and 30 min after addition of thrombin

Fibrin polymerization was monitored as the changes in the absorbance values over time at 595 nm. Thrombin preincubation with cyanidin, quercetin and silybin resulted in the inhibition of thrombin ability to induce fibrinogen polymerization, depending on their concentration (Fig. 1a–c). When thrombin was preincubated with cyanin, (+)-catechin and (−)-epicatechin and then added to fg the inhibitory effect of polymerization of human fibrinogen was not observed (Fig. 1d–f). Contrary to cyanin, (+)-catechin and (−)-epicatechin cyanidin in a dose-dependent manner reduced the initial velocity of fibrin polymerization; and at a concentration of 5 μM, total inhibition of thrombin activity was observed (Fig. 1a). Similar results were obtained for quercetin (Fig. 2b), but the concentration caused the total inhibition of thrombin activity to be ten times higher (50 μM) than in the case of cyanidin. Silybin also decreased in a dose-dependent manner the initial velocity of fibrin polymerization; however at the highest concentration (1,000 μM) used, complete inhibition of thrombin activity was not observed (Fig. 1c).

**Fig. 3:**
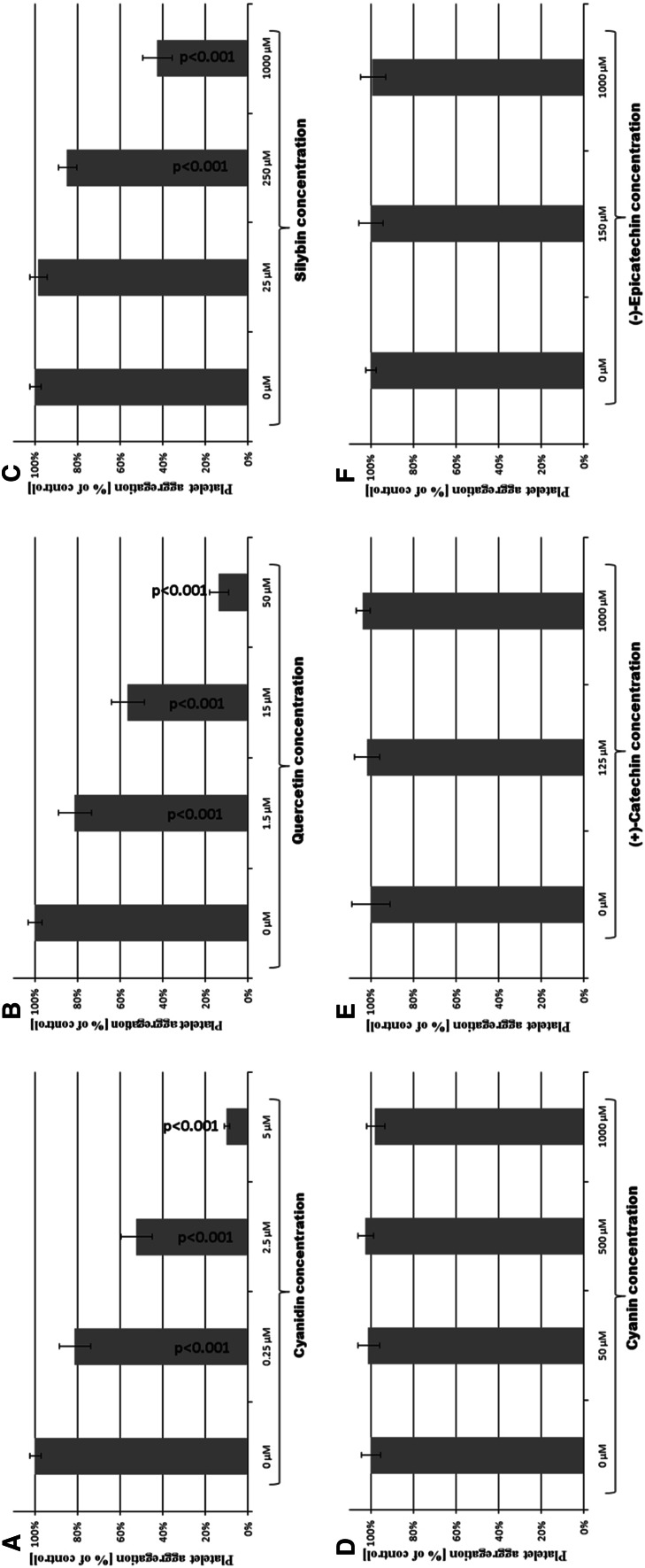
The effect of polyphenolic compounds [cyanidin, quercetin, silybin, cyanin, (+)-catechin and (−)-epicatechin] on the thrombin-induced platelet aggregation. Thrombin was preincubated with polyphenols at 37 °C for 10 min. Thrombin-catalyzed platelet aggregation was monitored for 10 min in the dual-channel Chrono-log aggregometer. The results are expressed as % of aggregation in comparison to the control samples (thrombin without tested compounds). Data represent mean ± SD of eight independent experiments done in duplicate

The electrophoretic patterns of fibrinogen under reducing conditions show the bands corresponding to Aα, Bβ and γ chains in the structure of this protein. Thrombin action on fibrinogen resulted in the disappearance of Aα and γ chains and appearance of additional bands corresponding to γ–γ chains, as well as high molecular weight α-polymers on the top of the gel (Fig. 2a). Preincubation of thrombin with cyanidin (2.5 μM) or quercetin (15 μM) significantly inhibited the formation of γ–γ chains and α-polymers, and inhibited the decay of bands corresponding to Aα and γ chains (Fig. 2b, c). The thrombin preincubation with cyanidin and with quercetin at IC_50_ concentration of amidolytic inhibition (0.25 μM for cyanidin and 1.5 μM for quercetin respectively) also inhibited the formation of γ–γ chains and α-polymers. However, after 15 min of the experiment, these bands corresponding to γ–γ chains and α-polymers appeared, while loss of bands corresponding to Aα and γ chains scarcely after 30 min was observed (Fig. 3b, c). SDS-PAGE of fg treated with thrombin preincubated with silybin showed that this polyphenolic compound slightly reduced the formation of γ–γ chains and α-polymers at concentration of the compound of 250 μM. After 15 min, the electrophoretic pattern was similar to the control (Fig. 2d). In the electrophoresis of fg treated with thrombin preincubated with cyanin, (+)-catechin or (−)-epicatechin, no changes were observed (Fig. 2e–g).

The exposure of thrombin to cyanidin or quercetin resulted in dose-dependent decrease of the ability of thrombin to induce platelets aggregation. Cyanidin at a concentration of 5 μM reduced aggregation to 10 % of control, while quercetin at a concentration of 50 μM reduced platelets aggregation to 4 % (Fig. 3a, b). Silybin effect on thrombin ability to induce platelet aggregation was also observed, but was much weaker when compared with cyanidin and quercetin, and at the concentration of 1,000 μM the aggregation reached 43 % of the control (Fig. 3c). Cyanin, (+)-catechin and (−)-epicatechin added to thrombin had no effect on thrombin ability to stimulate platelets aggregation (Fig. 3d–f).

### BIAcore analyses

**Fig. 4:**
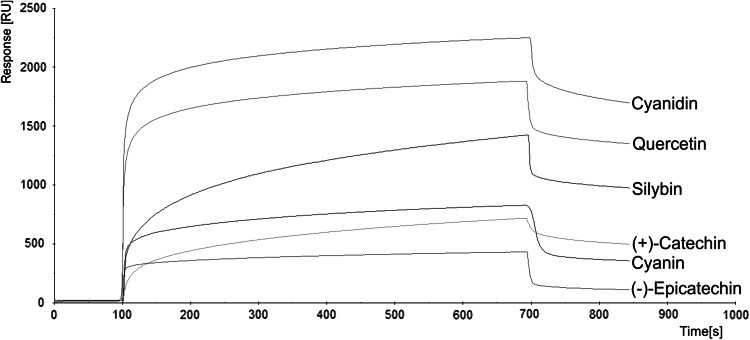
Overlay sensorgrams for SPR analysis of polyphenolic compounds [cyanidin, quercetin, silybin, cyanin, (+)-catechin and (−)-epicatechin] bound to human thrombin immobilized on CM5 sensor chip. Polyphenols were injected at a concentration of 1,000 μM to the channel with immobilized thrombin. Sensorgrams were collected using BIAcore system and BIAevalution software 3.1

The sensorgrams obtained in BIAcore analyses (Fig. 4) demonstrated that cyanidin and quercetin caused a strong response in the interaction with immobilized thrombin (2,251 RU for cyanidin and 1,882 RU for quercetin), while (+)-catechin, cyanin and (−)-epicatechin induced rather a low response to the interaction with this protein [827 RU for cyanin, 717 RU for (+)-catechin and 431 RU for (−)-epicatechin, respectively]. Response to silybin (1,424 RU) was higher than to (+)-catechin and (−)-epicatechin, but lower than cyanidin and quercetin.

The kinetic parameters obtained from the sensorgram analyses of the interaction of immobilized thrombin with polyphenolic compounds received using BIAcore system and BIAevaluation 3.1 software, presented in Table 2, show that cyanidin and quercetin association to thrombin was kinetically promoted (*k*_a_ for cyanidin is 85.6 M^–1^ s^–1^, and for quercetin is 43.2 M^–1^ s^–1^), whereas cyanin showed the lowest association rate (*k*_a_ = 0.95 M^–1^ s^–1^). Analyses of equilibrium constants demonstrate that the highest affinity to thrombin has cyanidin (*K*_A_ = 1.28 × 10^8^ M^–1^, *K*_D_ = 7.79 × 10^−9^ M) and quercetin (*K*_A_ = 2.59 × 10^7 ^M^–1^, *K*_D_ = 3.87 × 10^−8 ^M). Cyanin and (−)-epicatechin show the lowest affinity to thrombin (cyanin *K*_A_ = 115 M^–1^ and *K*_D_ = 8.63 × 10^−3 ^M, while (−)-epicatechin *K*_A_ = 192 M^–1^, *K*_D_ = 5.19 × 10^−3 ^M).

**Table 2:** Kinetic parameters of the thrombin interaction with polyphenolic compounds

Compound	RU	*k*_a_ (1/M s)	*k*_d_ (1/s)	*K*_A_ (1/M)	*K*_D_ (M)
Cyanidin	2,251	85.60	6.67 × 10^−7^	1.28 × 10^8^	7.79 × 10^−9^
Quercetin	1,882	43.20	1.67 × 10^−6^	2.59 × 10^7^	3.87 × 10^−8^
Silybin	1,424	7.11	1.32 × 10^−4^	5.39 × 10^4^	1.86 × 10^−5^
Cyanin	827	0.95	8.24 × 10^−3^	1.15 × 10^2^	8.63 × 10^−3^
(+)-Catechin	717	3.62	1.78 × 10^−4^	2.03 × 10^4^	4.92 × 10^−5^
(−)-Epicatechin	431	4.37	2.27 × 10^−2^	1.92 × 10^2^	5.19 × 10^−3^

The association rate (*k*_a_), the dissociation rate (*k*_d_), equilibrium association constants *K*_A_ and equilibrium dissociation constants *K*_D_ were obtained in BIAcore analysis (from 5 sensorgrams at the concentrations ranging from 50 to 1,000 μM) using BIAevaluation 3.1 software. Response (RU) was shown for maximum used concentration of the analyte (1,000 μM)

### Analysis of thrombin inhibition parameters

**Fig. 5:**
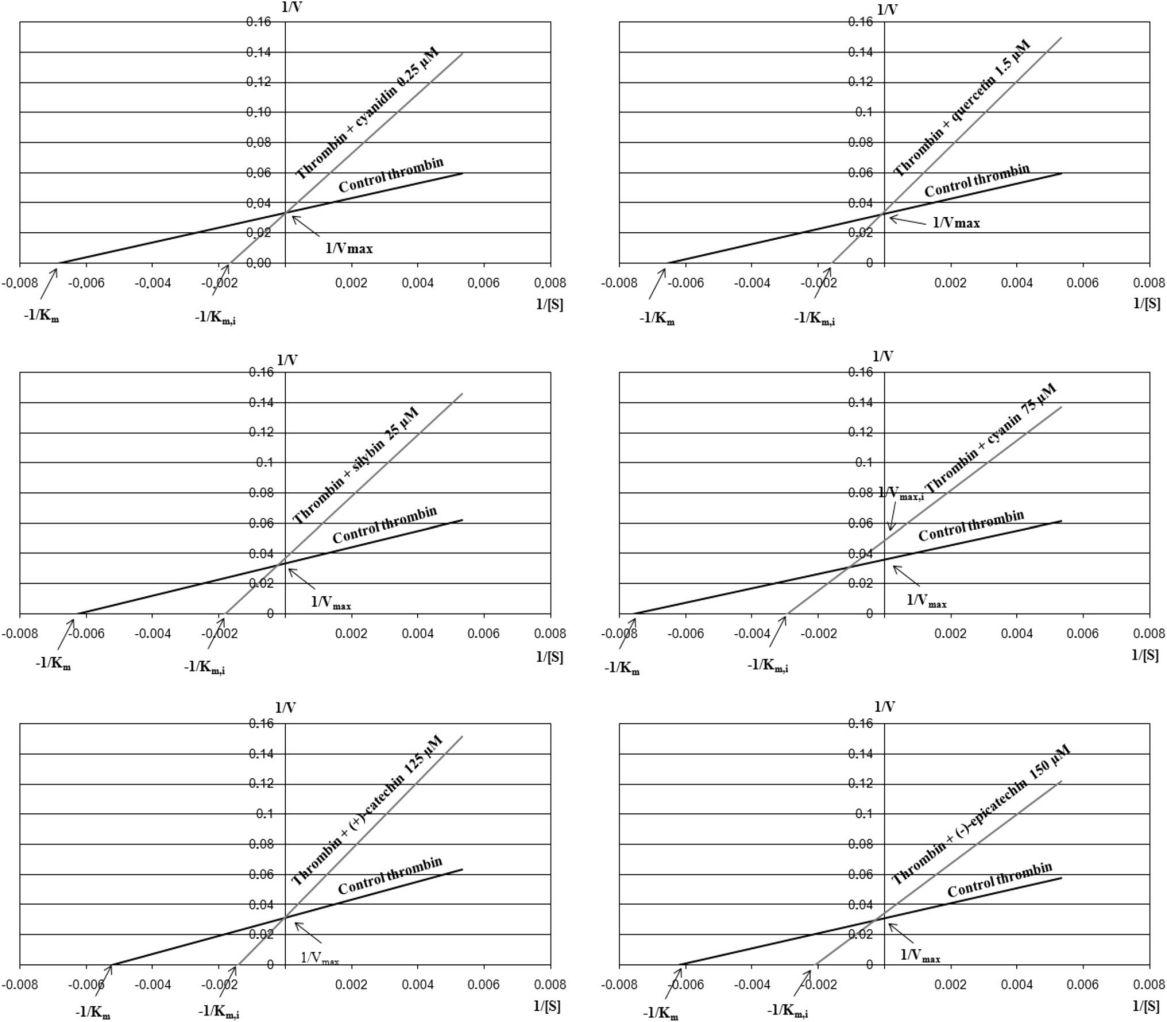
Lineweaver–Burk curves plotted for the control thrombin and thrombin incubated with polyphenolic compounds. Data represent curves for means of four independent experiments

The analysis of the kinetic parameters obtained from Lineweaver–Burk curves shows that cyanidin, quercetin, silybin, (+)-catechin and (−)-epicatechin (Fig. 5) act as competitive inhibitors. These compounds resulted in an increase in the Michaelis constant (*K*_m_) value, whereas the maximum speed (*V*_max_) of chromogenic substrate decomposition reaction by thrombin remained unchanged (Table 3). In the case of the Lineweaver–Burk curve (Fig. 5) for thrombin incubated with cyanin, we observed simultaneous decrease of the maximum speed and the increase of the Michaelis constant (Table 3). This indicates a mixed type of inhibition of thrombin amidolytic activity by this compound.

**Table 3:** Effect of polyphenolic compounds [cyanidin, quercetin, silybin, cyanin, (+)-catechin and (−)-epicatechin] on kinetic parameters of chromogenic substrate hydrolysis by thrombin

	*K*_m_ (10^−6 ^M)	*V*_max_ (10^−6^ mol/min)	*k*_cat_ (1/s)
Control	158.7	30.7	29.1
Cyanidin	600.6	30.3	28.7
Quercetin	633.8	29.4	27.8
Silybin	550.5	27	25.6
Cyanin	344.6	20.8	19.7
(+)-Catechin	700.1	31.2	29.5
(−)-Epicatechin	481.5	29.4	27.8

Parameters: Michaelis constant (*K*_m_) and maximum speed (*V*_max_) of reaction was obtained from Lineweaver–Burk curves; enzyme catalytic constant (*k*_cat_) was calculated from formula: *k*_cat_ = *V*_max_/*E*_0_

## Discussion

Polyphenols are probably the most investigated molecules of nutritional interest. Much research has shown the importance of antithrombotic effect of polyphenol-rich plant extracts (Chua and Koh, 2006). In our previous in vitro studies, we found that incubation with polyphenol-rich extracts from chokeberry and grape seeds resulted in the changes of coagulation properties of human plasma (Bijak *et al*., 2011). Moreover, we also observed that incubation of human thrombin, both with chokeberry and grape seeds extracts, caused the inhibition of amidolytic and proteolytic activity of this enzyme (Bijak *et al*., 2013b). The studied extracts are very rich sources of polyphenolic compounds (mainly from a flavonoid group) (Bijak *et al*., 2011). The anticoagulant effects of plant polyphenolic–polysaccharide conjugates from *Asteraceae* and *Rosaceae* families were demonstrated by Pawlaczyk et al. (2009), who presented that the polyphenolic-rich compounds from 17 different plants of *Asteraceae* and *Rosaceae* families prolonged the clotting time of human plasma. Pawlaczyk et al. (2011) also reported the inhibitory effect of polyphenolic–polysaccharide complex isolated from *Erigeron canadensis* L. on thrombin activity. According to that work, the inhibitory effect probably was dependent on the carbohydrate part of the complex and the effect on thrombin was mediated by heparin cofactor II. However, it was proven following the example of similar polyphenolic–polysaccharide glycoconjugates isolated from *Fragaria vesca* L. leaves (Pawlaczyk *et al.*, 2013) that if the glycoconjugate was richer in polyphenolic components, the in vitro anticoagulant effect was better. Inhibition of thrombin amidolytic activity by pomegranate fruit and grape seeds components was also reported (Cuccioloni *et al*., 2009b).

Polyphenolic compounds are a broad group of organic secondary plant metabolites having one or more aromatic rings in the molecule and containing from more than one to ten of hydroxyl, phenolic groups. Polyphenolic compounds have been classified into several groups, including hydroxybenzoic acids, hydroxycinnamic acids, coumarins, xanthones, stilbenes, antraquinones, lignans and flavonoids (Manach *et al*., 2005). The largest and best known group among the polyphenolic compounds are flavonoids. The basic skeleton of flavonoid molecule consists of 15 carbon atoms (formula C6–C3–C6) forming the two benzene rings (A- and B-ring), between which there is a three-carbon unit (C3) closed in the heterocyclic pyran or pyrone ring (C-ring). Flavonoids are divided into six subgroups: anthocyanins, flavanols, flavanones, flavones, flavonols and isoflavones (Ullah and Khan, 2008).

**Fig. 6:**
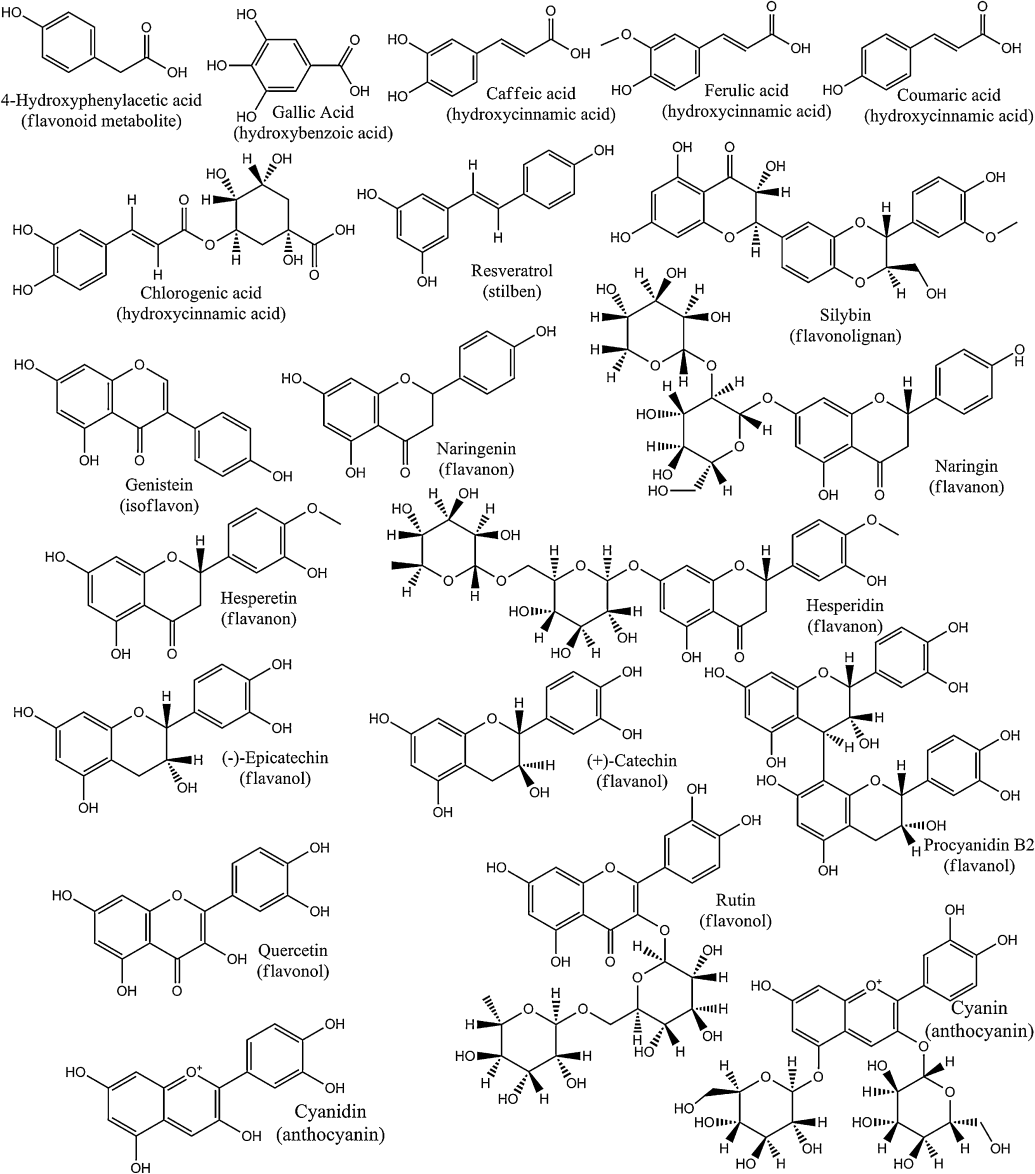
Chemical structures of polyphenolic compounds used in the study. Chemical formulas were downloaded from http://pubchem.ncbi.nlm.nih.gov/ as InChI. The visualization of chemical formulas was performed using ChemBioDraw Ultra Software from ChemBioOffice^®^ Ultra 12.0. suite

In our study we tested 20 polyphenolic compounds occurring most abundantly in nature and belonging to the main group of polyphenols (Fig. 6) at the highest used concentration of 1,000 μM. The results, presented in Table 1, demonstrate that of all polyphenolic compounds examined in this study, only six belonged to the flavonoid class [cyanidin, quercetin, silybin, cyanin, (+)-catechin and (−)-epicatechin] and had inhibitory effect on thrombin activity (the strongest effect showed cyanidin and quercetin). According to our observations, flavonoids which inhibit thrombin amidolytic activity belong to flavanols, flavonols anthocyanins (aglycones with –OH substituents at the position of R1 and R2 in the B-ring). Only silybin has a methoxy group at the R1 position. These results are consistent with data presented by Mozzicafreddo et al. (2006). They also reported that flavonoids showed an inhibitory effect on thrombin amidolytic activity. Jedinák et al. (2006) demonstrated that silybin and quercetin strongly inhibited thrombin’s ability to hydrolyze *N*-benzoyl-phenylalanyl-valyl-arginine-paranitroanilide (IC_50_ for silybin was 20.9 μM, and for quercetin 30.0 μM, respectively at 0.6 mM substrate concentration). In their study these flavonoids also showed very strong inhibitory effect on trypsin and urokinase amidolytic activity (for trypsin, silybin IC_50_ was 3.7 μM and quercetin IC_50_ was 15.4 μM, while for urokinase, silybin IC_50_ was 21.0 μM and quercetin IC_50_ was 12.1 μM). We also studied the effect of DMSO on thrombin activity at the same concentration as used in the case of polyphenolics dissolved in this solvent. After 5 % DMSO treatment, we did not observe any influence on thrombin activity.

The most important function of thrombin is its proteolytic activity against fibrinogen and platelet PAR receptors. Thrombin has much higher affinity to these molecules, than to smaller compounds such as the chromogenic substrate (Crawley *et al.*, 2007). There are no reports in the literature about the effects of polyphenolic compounds on thrombin proteolytic activity. To verify this effect, we chose compounds with distinct effects on the amidolytic activity of thrombin.

Fibrinogen is a glycoprotein with a molecular weight of 340 kDa, containing in its structure three pairs of different polypeptide chains called, respectively, Aα (610 aa, 67 kDa), Bβ (461 aa, 56 kDa) and γ (411 aa, 48 kDa). These chains are connected by 29 disulfide bonds forming a dimeric molecule (Aα Bβ γ)_2_ (Wolberg, 2007). Thrombin removes the N-terminal peptides from the Aα and Bβ chains which leads to fibrin formation. Thrombin also activates coagulation factor XIII which stabilizes the fibrin clot by catalysis of covalent bonds between γ chains in the D domains of adjacent fibrin monomers and formation of α-polymers (Bijak *et al*., 2013a; Muszbek *et al*., 1999).

Preincubation of thrombin only with three of six tested compounds changed the ability of thrombin to induce fibrinogen polymerization. We observed that only cyanidin, quercetin and silybin in a dose-dependent manner decreased the maximal velocity of thrombin-induced fibrinogen polymerization (Fig. 1a–c). When thrombin was preincubated with cyanin, (+)-catechin or (−)-epicatechin, the velocity of thrombin-induced fibrinogen polymerization was very similar to the velocity of fibrinogen polymerization induced by untreated thrombin (Fig. 1d–f). SDS-PAGE analysis (Fig. 2) confirmed the results obtained by spectrophotometric measurement of fibrinogen polymerization. In this analysis we used the polyphenolic compounds at concentrations equal to IC_50_ of thrombin amidolytic activity of each of them and ten times higher than these IC_50_ values, but not more than 1,000 μM.

Thrombin exosite I among others is responsible for binding to protease-activated receptors (PAR). Receptors PAR-1 and PAR-4 are present on the human platelet surface. Thrombin cleaves the N-terminal extracellular domain of PAR to expose a new N-terminus, which binds with the central extracellular loop of the same receptor causing its activation and initiating the intracellular signaling events (Hirano and Kanaide, 2003). Our study showed that exposure of thrombin to cyanidin, quercetin or silybin resulted in a decrease in thrombin ability to induce platelet aggregation (Fig. 3a–c). This experiment also confirmed that cyanin, (+)-catechin and (−)-epicatechin had no inhibitory effect on the proteolytic activity of thrombin (Fig. 3d–f). Both experiments with human fibrinogen and platelets demonstrated that cyanidin, quercetin and silybin inhibited thrombin proteolytic activity. Moreover, the inhibitory effect of silybin on thrombin was significantly weaker than the effect of cyanidin and quercetin. Asmis et al. (2010) suggest that 0.5 % DMSO inhibits platelet response to arachidonate, but aggregation in response to other agonists (ADP, collagen, ristocentin, epinephrine, U46619) was not affected by DMSO. We also checked the effect of 0.77 % DMSO on blood platelet activity and did not observe any changes in thrombin-induced aggregation between control and DMSO treatment platelets.

The next step of our study was to give a more detailed characterization of the interaction of thrombin with previous (due to their action) polyphenolic compounds. The BIAcore interaction analysis system may be used to examine the influence of the compounds on each other, i.e., on proteins, in terms of specificity of a binding reaction, kinetics and affinity. BIAcore analysis system uses surface plasmon resonance (SPR) to monitor the interaction between molecules during the experiment time (Torreri *et al*., 2005). In our analysis, among the tested compounds the highest affinity to thrombin was presented by cyanidin and quercetin (Table 2). These results are in agreement with BIAcore parameters obtained by Mozzicafreddo et al. (2006). They observed that quercetin has the lowest *K*_D_ value, whereas *K*_D_ for (−)-epicatechin was the highest. Similar parameters of silybin and (+)-catechin to association thrombin, despite their clearly distinct effect on the enzyme, are probably caused by the fact that, in BIAcore analysis, compounds bind to whole protein. When a ligand binds to the part of the protein which has no effect on its function in BIAcore, we observe the same response as in the case of binding to the enzyme active center. This suggests that (+)-catechin probably bind also to other places of the enzyme. Cyanidin and quercetin, in BIAcore analyses, show the strongest affinity to thrombin, which is probably even stronger than the fibrinogen and PAR receptors affinity. Therefore, it explains the inhibition of thrombin proteolytic activity caused by these compounds. Only the partial inhibition of thrombin proteolytic activity by silybin can be explained by the fact that silybin affinity to thrombin is higher than of cyanin, catechin or epicatechin, but lower in comparison to cyanidin and quercetin.

Analysis of graphs plotted by the Lineweaver–Burk linearization method (Lineweaver and Burk, 1934) (Fig. 5) demonstrated a competitive nature of human thrombin inhibition by using polyphenol aglycones. This means that these compounds mimic the structure of the substrate and reversibly interact with the free form of the enzyme in competition with the substrate for the enzyme active site. When the inhibitor occupies the active center of the enzyme, it prevents binding of the substrate and abolishes product generation. This inhibition may be reduced by adding more substrate to the reaction mixture (Bjelakovic *et al*., 2002). Our results obtained from Lineweaver–Burk curves confirm these assumptions (Table 3). Cyanidin, quercetin, silybin, (+)-catechin and (−)-epicatechin caused an increase of Michaelis constant value, while no effect on the maximum speed of reaction and on the enzyme catalytic constant was observed. Only in the case of cyanine we observed a mixed type of inhibition. This is probably due to the presence of glycoside residues in the compound, which non-specifically interact with enzyme or chromogenic substrate.

The molecular docking performed by Liu et al. (2010) demonstrated that flavonoids due to binding to the thrombin active center might block its activity. They also reported that more –OH groups in the B-ring of a flavonoid structure would increase thrombin inhibition by polyphenolic compounds. It could suggest an important role of these groups in the interaction with a catalytic triad. Similar experiments were presented by Shi et al. (2012). Their results showed that 3′-hydroxyl group and 4′-hydroxyl group in the B-ring of a flavonoid structure, as well as 3-hydroxyl rest in the C-ring of it, were very important for the inhibition of thrombin activity. Li et al. (2012) docking studies showed that the B-ring and C-ring in flavonoids may interact well with S1 pocket and S2 pocket of thrombin, respectively. A-ring only partly interacts with the S3 pocket in the thrombin molecule. We also reported that 3′-hydroxyl group and 4′-hydroxyl group in the B-ring of a flavonoid played a very important role in thrombin inhibition. Probably, these groups form hydrogen bonds with amino acids forming S1 pocket, which means that B-ring with hydroxyl groups at the position of R1 and R2 may imitate arginine residue in P1 of the thrombin substrate.

Our present study for the first time comprehensively analyzes the mechanism of thrombin inhibition caused by the selected natural occurring polyphenolic compounds and shows that not all examined structures that inhibit amidolytic activity of thrombin may block its proteolytic activity. We demonstrate that cyanidin and quercetin have the strongest inhibitory effect on thrombin activity. These polyphenolic compounds might be potential structural bases and source to find and project nature-based, safe, orally bioavailable direct thrombin inhibitors. However, it is known that the studied plant polyphenolic compounds can hardly reach therapeutic concentrations in vivo, because their bioavailability in the digestive tract is not high. Polyphenol compounds can also bind with many components of blood plasma (mainly by albumin) and the real effect of these compounds on coagulation may be mediated also by a different mechanism than their action on thrombin. Mozzicafreddo et al. (2006) showed that quercetin had an anti-clotting effect (prolonged thrombin time) at a concentration of 100 μM and higher. But our studies suggest that cyanidin and quercetin molecular structures could be used as pharmacophores to design and synthesize substances with more accessible and more specific inhibitory properties. The next step of research should include chemical modifications of cyanidin and quercetin structure to choose the best compounds for future drug designs.
